# Neonatal Dyshormonogenetic Goiter with Hypothyroidism Associated with Novel Mutations in Thyroglobulin and SLC26A4 Gene

**DOI:** 10.3390/pediatric13020029

**Published:** 2021-05-02

**Authors:** Valeria Calcaterra, Rossella Lamberti, Claudia Viggiano, Sara Gatto, Luigina Spaccini, Gianluca Lista, Gianvincenzo Zuccotti

**Affiliations:** 1 Pediatric and Adolescent Unit, Department of Internal Medicine, University of Pavia, 27100 Pavia, Italy; 2 Pediatric Unit, Department of Pediatrics, “Vittore Buzzi” Children’s Hospital, 20154 Milan, Italy; rossella.lamberti@unimi.it (R.L.); claudia.viggiano@unimi.it (C.V.); gianvincenzo.zuccotti@unimi.it (G.Z.); 3 Neonatal Pathology and Neonatal Intensive Care Unit, Department of Pediatrics, “V. Buzzi” Children’s Hospital, University of Milan, 20154 Milan, Italy; sara.gatto@asst-fbf-sacco.it (S.G.); gianluca.lista@asst-fbf-sacco.it (G.L.); 4 Clinical Genetics Unit, Department of Obstetrics and Gynecology, “V. Buzzi” Children’s Hospital, University of Milan, 20154 Milan, Italy; luigina.spaccini@asst-fbf-sacco.it; 5 Department of Biomedical and Clinical Science “L. Sacco”, University of Milan, 20157 Milan, Italy

**Keywords:** congenital goiter, primary congenital hypothyroidism, thyroid dyshormonogenesis, newborn

## Abstract

Congenital goiter is an uncommon cause of neck swelling and it can be associated with hypothyroidism. We discuss a case of primary hypothyroidism with goiter presenting at birth. Ultrasound showed the enlargement of the gland and thyroid function tests detected marked hypothyroidism. Genetic analysis via next generation sequencing (NGS) was performed finding two mutations associated with thyroid dyshormonogenesis: c.7813 C > T, homozygous in the exon 45 of the thyroglobulin gene (TG) and c.1682 G > A heterozygous in exon 15 of the SLC26A4 gene (pendrin). Sanger sequencing of parents’ DNA samples revealed that the first mutation (c.7813 C > T) was inherited from both of them, while the second one (c.1682 G > A) was inherited from the mother. Hormone replacement therapy was started, following which a gradual decrease in the size of the goiter was seen with the normalization of hormonal levels. Normal infant growth status and neurological development were recorded during follow-up. Neonatal dyshormonogenetic goiter with hypothyroidism may represent an unusual cause of neonatal neck mass. Early identification and hormone replacement therapy are crucial for a better neurodevelopmental outcome. Genetic analysis is mandatory in order to reach a specific diagnosis and to elucidate new patterns of thyroid disorder.

## 1. Introduction

Congenital goiter is a rare clinical condition and uncommon cause of neck swelling, characterized by the enlargement of the thyroid gland with a nodular or diffuse pattern [1].

Goiter development depends on cell infiltration and thyroid follicular cell proliferation secondary to hypertropinemia and inadequate thyroid hormone production [2]. 

The differential diagnosis of congenital goiter includes hemangioma/lymphangioma, teratoma, hamartoma, cystic hygroma, ectopic thymus, branchial cleft cysts, thyroglossal duct cyst, cervical neuroblastoma and meningoceles [3,4]. 

In most cases, a congenital goiter is acquired; this can be caused by in utero deficiency or excessive exposure to iodine, antithyroid medications or other goitrogens, transplacental antithyroid maternal antibodies in Graves’ disease, and inappropriate maternal hormone replacement therapy. Congenital hypothyroidism (CH) [1,3,5] due to the impairment of the thyroid hormones biosynthesis (dyshormonogenesis) can also occur, albeit rarely. In particular, the loss-of-function mutations of genes involved in thyroid hormogenesis leads to impaired hormonal synthesis with or without compensatory goiter. Mutations may affect thyroglobulin (TG), thyroid peroxidase (TPO), dual oxidase 2 (DUOX2) and its associated protein (DUOXA2), sodium-iodide symporter (SLC5A5), apical iodide transporter pendrin (SLC26A4) and iodotyrosine deiodinase (IYD) [6,7]. 

We describe a case of neonatal dyshormonogenetic goiter with hypothyroidism, associated with novel mutations in the TG and SLC26A4 gene.

## 2. Case Report

The patient was transferred to the Neonatal Intensive Care Unit of the Vittore Buzzi Children’s Hospital, Milan, about eleven hours after birth, due to the presence of a bilateral anterolateral cervical mass.

The boy was born by vaginal delivery at 38 weeks and 3 days gestational age. Amniotic fluid was clear and maternal infectious risk factors were not found. The pregnancy was complicated by gestational diabetes treated with diet therapy and autoimmune hypothyroidism (with anti-thyroid peroxidase antibodies positivity) in replacement therapy. 

The infant’s parents were from Egypt and were consanguineous. There was a family history of three paternal uncles who underwent surgery for cervical mass during childhood. Fetal ultrasound at 33 weeks of gestational age was normal, without mass in the fetal neck.

Birth weight was adequate for gestational age (3250 g). Length and head circumference were 51 cm and 34 cm, respectively. The first and fifth minute Apgar scores were 10–10.

**Figure 1:**
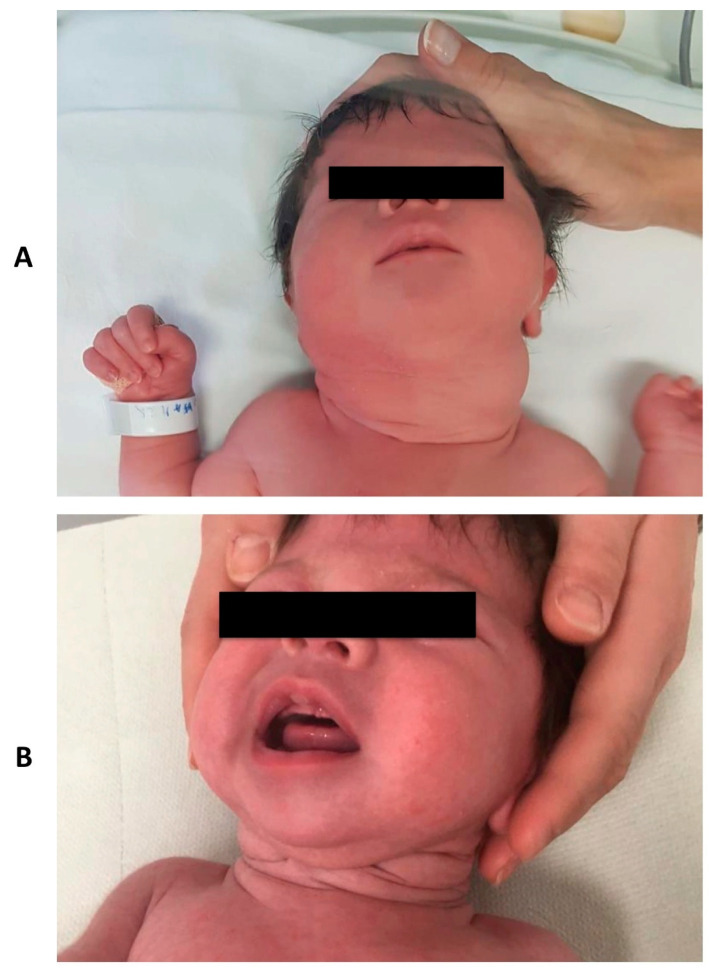
Clinical aspect of the neck mass at birth (Panel **A**) and at three months follow-up (Panel **B**).

On first clinical examination, a remarkable cervical swelling was observed (Figure 1A). No signs of respiratory distress or other clinical malformations were seen. 

**Figure 2:**
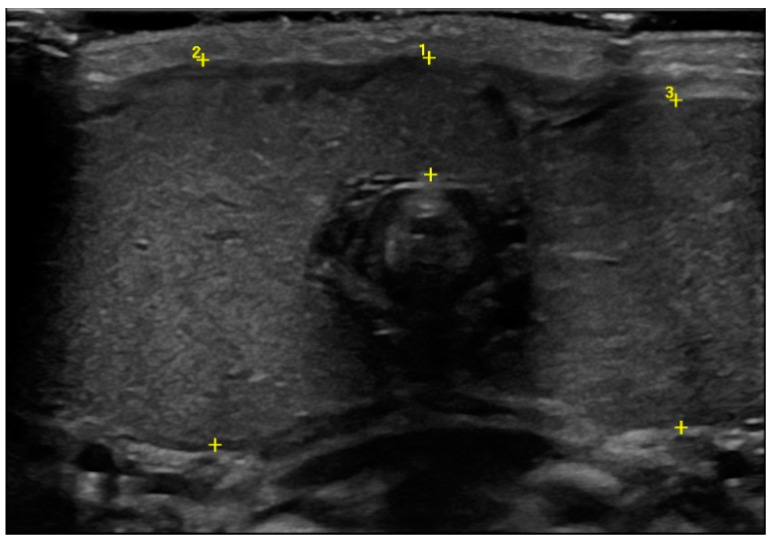
Thyroid ultrasound at birth in which the enlargement of the gland was detected (cursor 1 isthmus, 0.8 cm; cursor 2 antero–posterior diameter of the right lobe, 2.5 cm; cursor 3 antero–posterior diameter of the left lobe, 2.2 cm).

Ultrasound showed an enlargement of the thyroid gland (antero–posterior diameter of the right lobe was 2.5 cm and the left one was 2.2 cm, Figure 2), with a normal echogenic pattern and increased blood flow with central vascularization. 

The endocrinological profile showed increased TSH levels (TSH > 100 mIU/L, nv 1–6.5) with low levels of thyroid hormones (FT3 6.6 pmol/L nv 4.1–8.1; FT4 6.4 pmol/L vn 10.3–19.9), TG < 0.04 ng/mL (nv < 78). 

The laboratory assessment of autoantibodies against thyroglobulin (Tg), thyroid peroxidase (TPO) and thyroid-stimulating hormone receptor (TSH-R) was negative. Urinary iodine level was in the normal range. Radiological assessment of the knee showed the ossification nucleus of femoral epiphysis (diameter 3.9 mm) but the absence of the tibial one. Hearing screening results were normal.

Immediately after the diagnosis was made, oral levo-thyroxine was started at a dosage of 8 ug/kg per day. The infant’s growth was regular, with effective suction during breastfeeding. Objective neurological assessments were always within the normal range. 

Genetic analysis via the next generation sequencing of causative genes associated with congenital hypothyroidism (DUOX2, DUOXA2, FOXE1, GLIS3, IYD, NKX2-1, NKX2-5, JAG1, PAX8, SLC5A5, SLC26A4, TG, TPO, TSHR), was performed using DNA extracted from a blood sample. 

Two mutations were detected: c.7813 C > T (p.Arg2605Stop) homozygous mutation in the exon 45 of TG gene (NM_003235, Chr8(GRCh37):g.134128911C > T) and c.1682 G > A (p.Gly561Asp), heterozygous mutation in the exon 15 of SLC26A4 gene (NM_000441Chr7(GRCh37):g.107340595G > A). Sanger sequencing of parents’ DNA samples revealed that the first mutation (c.7813 C > T) was inherited from both parents, while the second one (c.1682 G > A) was inherited from the mother.

At the beginning of the second month of life, thyroid ultrasound showed a drastic reduction in the antero–posterior diameters of the lobes (1.3 cm), with a normal echogenic pattern. 

During follow-up, TSH and hormone levels were monitored and dosage was adjusted according to weight and hormone levels. The infant’s growth status and neurological development were regular. A progressive decrease in the size of the goiter was observed (Figure 1B).

## 3. Discussion

Congenital goiter presenting in the neonatal period is very rare and represents an uncommon cause of neck mass. Usually newborns have no symptoms; however, the development of complications due to tracheal compression inducing stridor, cyanosis or respiratory distress cannot be excluded [8]. 

In infants with congenital goiter, thyroid function tests should be performed. In addition, thyroid antibody tests should be carried out, depending on the results of hormonal levels and on the potential for maternal thyroid disorder (antithyroid medications, maternal excess iodine ingestion, maternal Graves’ disease). Thyroid antibody tests should be negative in the case of an infant with hypothyroidism and goiter that results from an inborn error of thyroid hormone metabolism (dyshormonogenesis) [9]. Moreover, the measurement of 24-hour/spot urinary iodine in the mother and/or infant can be used to exclude excess iodine exposure [10]. 

In the congenital goiter, color-doppler ultrasonography is useful in gaining information on the position and dimension of the thyroidal lobes and the vascularization pattern (peripheral increased flow is suggestive of a hypothyroid goiter, while central vascularization is indicative of a hyperthyroid goiter) [1,4]. 

In our case, the neck swelling of the newborn was clinically compatible with the goiter. The diagnostic suspicion was confirmed by ultrasound, showing the hypertrophy of the thyroid gland. In addition, thyroid function tests detected marked primary hypothyroidism, with a relatively high serum FT3 levels compared to FT4 levels [11]. Thyroid antibody tests were negative and urinary iodine levels were normal. Based on these results, we made the diagnosis of CH. 

CH is the most common cause of preventable intellectual disability. Early identification from neonatal thyroid screening programs and replacement therapy with levothyroxine are crucial for better neurodevelopmental and clinical outcomes [9]. 

Management depends on thyroid function and the identification of the underlying cause. If inborn errors of thyroid hormone production are suspected, genetic analysis may help to identify the specific diagnosis [2,12].

In our newborn, two novel mutations were detected: c.7813 C > T, homozygous, in the exon 45 of TG gene and c.1682 G > A, heterozygous, in the exon 15 of SLC26A4 gene.

Thyroglobulin is a glycoprotein secreted through the apical surface of the thyroid follicular cells into the colloid. TG gene mutations induce low or absent levels of serum Tg, elevated TSH levels, and low levels of circulating thyroid hormones [13]. CH due to TG gene mutation is inherited in an autosomal recessive manner. Therefore, patients typically have homozygous (as in our infant) or compound heterozygous gene mutations [14]. The incidence of an inborn error of thyroid hormogenesis associated with a TG gene mutation is 1:100,000 and more than 100 mutations of the human TG gene have been identified [15,16]. 

SLC26A4 (pendrin) is an anion exchange transport expressed in the ear, kidney and at the apical membrane of thyrocytes [15]. The biallelic recessive mutation induces Pendred syndrome, characterized by goiter, sensorineural deafness/hearing impairment with or without hypothyroidism [15,16,17]. 

The mutations found in our patient have never been reported in the literature or in the databases consulted (NCBI 1000 Genomes Browser, HGMD 2020.3, ClinVar) and they have been classified as Variants of Uncertain Significance. Dyshormonogenetic goiter is infrequently associated with gestational autoimmunity referred to as Hashimoto thyroiditis, as in our case. More frequently, a neonatal goiter is associated with maternal gestational autoimmune hyperthyroidism in replacement therapy [18]. As the TG mutation is homozygous, it could directly affect the baby’s clinical phenotype. Additional histological features could be used to distinguish the hypothyroidism forms including dyshormonogenetic goiter and autoimmune thyroiditis [19,20]. 

In addition, the association with the heterozygous mutation of SLC26A4 gene could contribute to the dyshormonogenesis. In fact, the association of rare variants not individually associated to an overt phenotype should explain the pathogenesis of CH. The typically sporadic presentation of CH can be related to the different inheritance patterns, variable expressivity and penetrance of this endocrinological disease [17].

## 4. Conclusions

Neonatal dyshormonogenetic goiter with hypothyroidism may represent an unusual cause of neonatal neck mass. Early identification and hormone replacement therapy are crucial for a better neurodevelopmental outcome. Genetic analysis is mandatory in order to reach a specific diagnosis. 

## Data Availability

The data presented in this study are available on request from the corresponding author.
